# FMAP: Functional Mapping and Analysis Pipeline for metagenomics and metatranscriptomics studies

**DOI:** 10.1186/s12859-016-1278-0

**Published:** 2016-10-10

**Authors:** Jiwoong Kim, Min Soo Kim, Andrew Y. Koh, Yang Xie, Xiaowei Zhan

**Affiliations:** 1 Department of Clinical Sciences, Quantitative Biomedical Research Center, University of Texas Southwestern Medical Center, 5323 Harry Hines Boulevard, Dallas, TX 75390 USA; 2 Harold C. Simmons Comprehensive Cancer Center, University of Texas Southwestern Medical Center, 5323 Harry Hines Boulevard, Dallas, TX 75390 USA; 3 Department of Bioinformatics, University of Texas Southwestern Medical Center, 5323 Harry Hines Boulevard, Dallas, TX 75390 USA; 4 Department of Pediatrics, University of Texas Southwestern Medical Center, 5323 Harry Hines Boulevard, Dallas, TX 75390 USA; 5 Department of Microbiology, University of Texas Southwestern Medical Center, 5323 Harry Hines Boulevard, Dallas, TX 75390 USA; 6 Center for the Genetics of Host Defense, University of Texas Southwestern Medical Center, 5323 Harry Hines Boulevard, Dallas, TX 75390 USA

## Abstract

**Background:**

Given the lack of a complete and comprehensive library of microbial reference genomes, determining the functional profile of diverse microbial communities is challenging. The available functional analysis pipelines lack several key features: (i) an integrated alignment tool, (ii) operon-level analysis, and (iii) the ability to process large datasets.

**Results:**

Here we introduce our open-sourced, stand-alone functional analysis pipeline for analyzing whole metagenomic and metatranscriptomic sequencing data, FMAP (*F*unctional *M*apping and *A*nalysis *P*ipeline). FMAP performs alignment, gene family abundance calculations, and statistical analysis (three levels of analyses are provided: differentially-abundant genes, operons and pathways). The resulting output can be easily visualized with heatmaps and functional pathway diagrams. FMAP functional predictions are consistent with currently available functional analysis pipelines.

**Conclusion:**

FMAP is a comprehensive tool for providing functional analysis of metagenomic/metatranscriptomic sequencing data. With the added features of integrated alignment, operon-level analysis, and the ability to process large datasets, FMAP will be a valuable addition to the currently available functional analysis toolbox. We believe that this software will be of great value to the wider biology and bioinformatics communities.

**Electronic supplementary material:**

The online version of this article (doi:10.1186/s12859-016-1278-0) contains supplementary material, which is available to authorized users.

## Background

Recent microbiome studies have revealed the complex functional relationships between microorganisms and their environment. Most notably, numerous human microbiome studies have aimed to elucidate the biological functional roles that microbial communities play within the niches of the human body, all of which can modulate host metabolism, development and health. Two large studies, the Human Microbiome Project [1] and the MetaHIT Consortium [2], have catalogued the various microbial communities found in the human body and further facilitated understanding of the relationship between changes in the human microbiome and the state of human health. These studies have utilized microbial taxonomic group classification using 16S rRNA sequences and gene content using whole genome shotgun (WGS) sequencing in order to identify the functional capabilities of microbial communities. Expression, translation and enzymatic functions have also been interrogated using techniques such as metatranscriptomics, metaproteomics and meta-metabolomics in order to understand how genomic composition translates into phenotype.

Functional characterization of microbiomes using sequencing by WGS and metatranscriptomics relies on (a) sensitive and accurate sequence alignment, (b) a functionally well-characterized sequence database, and (c) robust downstream statistical analysis for comparative and enrichment analysis. Currently, there are a few available software packages for metagenomics/metatranscriptomic functional characterization. MG-RAST [3] provides an easy-to-use web-interface for metagenomics analysis, including alignment, but imposes file size limits for users. HUMAnN [4] and MEGAN [5] both lack an integrated alignment tool and are notably unable to perform comprehensive downstream processes such as operon-level analysis [6]. Finally, ShotgunFunctionalizeR [7] is a package for determining statistically significant differentially abundant pathways, requiring pre-processed data in the form of raw COG (counts of orthologs) counts.

Therefore, we developed a tool called *F*unctional *M*apping and *A*nalysis *P*ipeline (FMAP). FMAP is a downloadable integrated package that utilizes raw sequencing data and sample information to perform advanced statistical analyses to identify differentially abundant features. FMAP can take raw sequence data and generate the following output: (i) an alignment of reads to a reference database, (ii) the abundances of gene families, and (iii) enriched operons and pathways from the differentially abundant (DA) gene analysis. Additionally, FMAP provides a customized comprehensive reference for metagenomics analysis and integrates a powerful suite of statistical and annotation modules, which can help the flexibility of functional analysis of metagenomics and metatranscriptomics data.

## Implementation

**Fig. 1:**
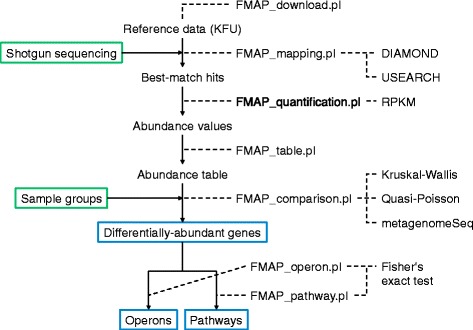
FMAP Workflow. A representation of the FMAP workflow, where input data is expected to be sequence data and the final output of each step includes alignments to the KFU reference database, quantification of KEGG ortholog groups (KOs), differentially abundant genes and operon and pathways enriched in the DA KOs. The green box indicates the input of the workflow and the blue box is the output result

FMAP performs sequence alignment, gene family abundance calculations, and differential feature statistical analysis (Fig. 1). The low-quality sequence reads and human sequences are first removed using BMTagger [8]. FMAP aligns the remaining reads using USEARCH [9] or DIAMOND [10] against a KEGG Filtered UniProt [11] reference cluster (KFU, see Additional file 1: Figure S2 for details), which is filtered for bacteria, fungi and archaea sequences with KEGG functional classifications in order to reduce the search library, yet retains functionally informative sequences (1,995,269 coding sequences). Best-hit matches are filtered by e-value < 1e-3, percent identity > 80 %. In sequencing data analysis, quantifying gene abundance by RPKM (reads per kilobase per million, which normalizes gene abundance by the length of genes) has become standard practice. However, because genes are comprised of promiscuous protein domains (e.g., binding domains in proteins with many different catalytic domains), the calculation of an accurate gene length is not trivial. Therefore, FMAP calculates the abundance of KEGG Orthologous groups (KOs) by (a) simply calculating the number of reads mapping to each KO (raw count) or (b) calculating RPKM where we assume that the gene length is the minimum gene length for the best hit (s). The RPKM value of the gene $g$, $RPKM(g)$, is calculated by employing the equation:

$$ RPKM(g)={\displaystyle \sum_{r\in R(g)}}\frac{1}{PL(r)\cdot 3}\times \frac{1}{T}\times {10}^9 $$

where $R(g)$ is the set of all sequencing reads mapped to the gene $g$, $PL(r)$ is the length of the best hit protein of the read $r$, and $T$ is the total number of mapped reads.

FMAP provides analysis of differentially abundant (DA) genes and enrichment analysis of pathways and operons, offering three built-in statistical testing methods to choose from: (1) metagenomeSeq [12], using the raw count data; (2) Kruskal-Wallis rank-sum tests (default), using RPKM; and (3) quasi-Poisson [7], also using RPKM. Since each of these three popular statistical methods has its own distinct advantages (e.g., metagenomeSeq is suited to modeling very sparse data, the Kruskal-Wallis rank-sum test has good performance in general, and quasi-Poisson has intermediate performance), they are all supported by FMAP and can complement each other in practical analysis. For example, the default method, the Kruskal-Wallis test, has robust performance and relatively high statistical power in a wide range of scenarios [12].

In order to assess differential operon abundance, we made the assumption that if genes in the same operon were differentially abundant, then the operon itself would be differentially abundant. In FMAP, an operon is a group of closely related orthologous genes (e.g., KO genes), although they do not necessarily come from one genera or species. We modeled the “operon” (defined by bioinformatics databases) as a smaller analysis unit compared to the pathway. Once we determined the list of differentially abundant KOs, we used the operon database (ODB3) [13] to identify operons in which all of the members were differentially abundant. Enrichment analysis was then used [14, 15] to determine DA operons and pathways using Fisher’s exact test, using the DA KOs which users can choose to filter on the raw *p*-value or FDR-adjusted *p*-value. The package reports the average log2 (fold change) and enrichment significance.

**Fig. 2:**
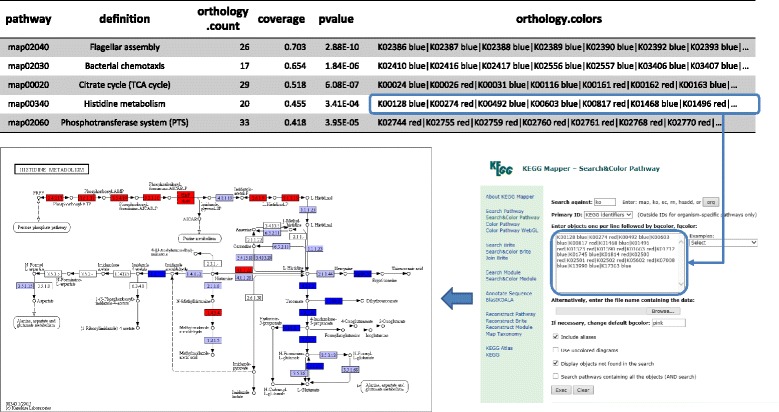
Integration of FMAP with KEGG pathway mapping visualization tool. Workflow for extracting enriched pathways and visualizing these pathways using the KEGG pathway-mapping tool available at http://www.genome.jp/kegg/tool/map_pathway2.html. Orthology.count: number of KO orthologies in the pathway; orthology.colors: FMAP outputs this column to highlight over-abundant (*red*) or under-abundant (*blue*) genes

In addition to the ability of FMAP to examine gene content (metagenomic) and expression data (metatranscriptomic), the software provides the output necessary to easily visualize results. From the orthology abundance results, users can generate heatmaps with the abundances of KO in samples. From the pathway enrichment analysis results, users can directly use the “orthology.colors” column as the input to the KEGG online pathway map tool: http://www.genome.jp/kegg/tool/map_pathway2.html (Fig. 2). This allows users to easily visualize pathways.

## Results

**Fig. 3:**
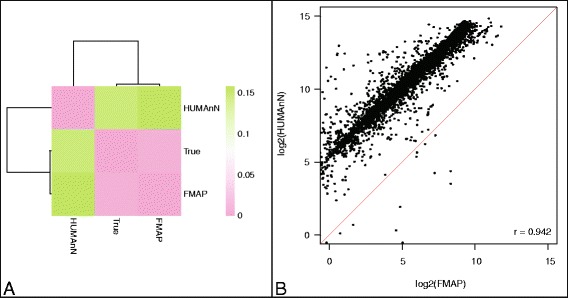
Performance of KO abundance calculation of FMAP and HUMAnN in simulated dataset (Illumina reads). **a** Heatmap of correlation distances between the true expected, FMAP-predicted and HUMAnN-predicted KO abundances in a simulated dataset (Illumina reads). **b** Plot of FMAP log2 KO abundance calculated from FMAP compared to log2 KO abundance calculated from HUUMAnN. r is Pearson’s correlation coefficient

FMAP has been applied to a broad range of datasets, with specific details included in the relevant sections below. But for the purposes of validating the accuracy of the software, we first compared the gene abundance results generated by FMAP to those generated by the well-established HUMAnN pipeline. We generated 2 simulated datasets of metagenomic sequences from 2,785 whole bacterial genomes. These datasets were generated at 1X coverage to resemble typical read length from the Illumina (96,258,884 reads, 100 bp) and 454 platforms (33,916,240 reads, average 227 bp) generated using ART [16]. Next we aligned the sequences using DIAMOND (a popular high-throughput aligner program, version 0.7.10) against FMAP’s KFU database and HUMAnN KEGG (v54) database. Then we generated the KO abundance calculation using (a) FMAP with the KFU best-hit match results and (b) HUMAnN with the KEGG best-hit match results. In order to determine the true abundance, we determined the KO abundance of the genes in the 2,785 bacterial genomes by aligning the genes to the KEGG protein database (v71) using DIAMOND. Compared to the true abundances and measured by correlation distance, RPKM abundances calculated by FMAP were much more similar to the true abundance than the abundances calculated by HUMAnN (Fig. 3a). When we compared the resulting abundances of FMAP and HUMAnN directly (Fig. 3b), the KO abundances shared a strong correlation (Pearson’s correlation coefficient, *r* = 0.942, *p*-value < 1x10^−6^). The result based on the 454 platform synthetic reads showed similar results (Additional file 2: Figure S1).

**Fig. 4:**
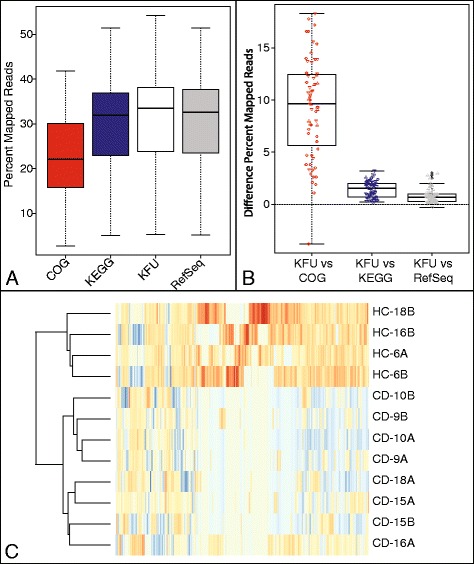
Performance of FMAP in real datasets. **a** Boxplots of percent mapping rates of FMAP using DIAMOND using four reference libraries, including the following: COG (2003) used by ShotgunFunctionalizeR, KEGG (v54) used by HUMAnN, KFU used by FMAP, and RefSeq (07/2014) used by MEGAN. **b** Boxplots of differences in mapping rates of COG, KEGG and RefSeq compared to the KFU. The values are the percent of reads mapped by KFU that are greater than the comparison database shown in A. The points indicate the values for samples from SRP002423 (▲), SRP050543 (▼), and SRP044400 (●). Boxplots drawn to represent the 25th and 75th percentiles (the lower and upper quartiles, respectively) as a box with a band in the box representing 50th percentile (the median). The upper whisker is located at the ‘smaller’ of the maximum x value and 3rd quartile + 1.5 inner quantile range (IQR), whereas the lower whisker is located at the ‘larger’ of the smallest x value and 1st quartile - 1.5 IQR. **c** Heatmap of differentially abundant genes of SRP002423. The samples were clustered by the genes into the two groups, healthy control (HC) and Crohn’s disease (CD)

To evaluate the performance of FMAP on different sequencing platforms and from different physiologic settings for metagenomics and metatranscriptomic data, we ran FMAP on a set of 103 publicly available samples collected from four microbial datasets generated using 454 and Illumina sequencing technology: (1) SRP002423, a gut metagenomic twin pair study examining biomarkers in Crohn’s Disease (454) [17]; (2) SRP000109, an ocean metagenomic sequencing study examining microbial communities at different sea depths (454) [18]; (3) SRP050543, an oral metatranscriptomic study examining biomarkers of biofilms in root caries (Illumina) [19]; and (4) SRP044400, a gut metagenomic study examining biomarkers in schizophrenia (Illumina) [20]. In evaluating the reference database customized in FMAP software, we aligned reads to the KEGG Filtered UniProt reference cluster (KFU) using DIAMOND. For comparison with the other commonly used reference databases, we used DIAMOND to map against three commonly used reference databases: (1) COG (2003); (2) RefSeq (07/2014); and (3) KEGG (v54) used in HUMAnN. Mapping rates were consistently higher using the KFU databases compared to other functional databases (Fig. 4a), with the median difference in percentage reads mapped 9.65 % higher than COG, 0.65 % higher than RefSeq and 1.55 % higher than KEGG (Fig. 4b).

**Fig. 5:**
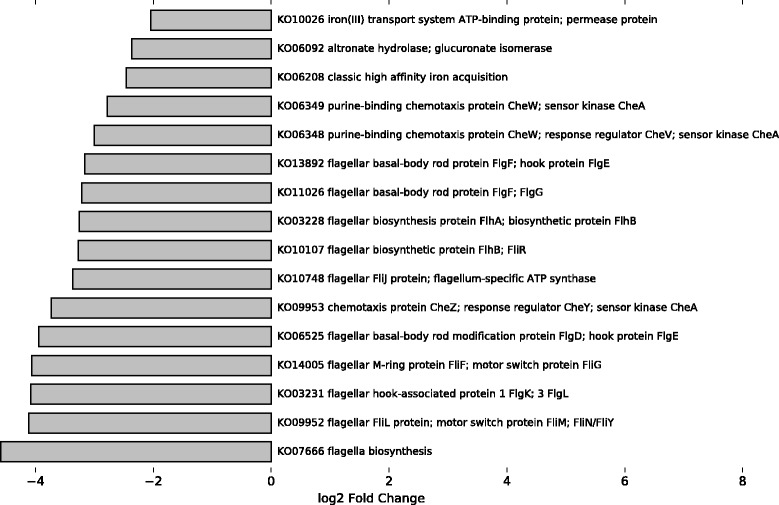
FMAP of differentially abundant operons. The FMAP analysis of a gut metagenomic twin pair study examining biomarkers in Crohn’s Disease (SRP002423) detected 16 differentially-abundant operons involved in the pathways of flagellar assembly, bacterial chemotaxis, ABC transporters, and pentose and glucoronate interconversions

To compare the performance of FMAP with comparable tools for pathway analysis, we performed functional analysis using (a) FMAP using the KFU alignment, (b) HUMAnN using the KEGG alignments and (c) ShotgunFunctionalizeR using raw counts of COGs calculated from the COG alignments. In this comparison, we used the Crohn’s twin study that includes 6 twin pairs (4 healthy controls and 8 Crohn’s Disease patients), where 2 twin pairs are phenotype discordant. For FMAP, the Kruskal-Wallis rank-sum test was used to detect DA genes, which resulted in 349 DA KOs (raw *p*-value < 0.05 and log2 (FC) > $\pm$ 1). The resulting DA KO profile differentiated the Crohn’s disease and healthy control samples, even in discordant twin pairs (Fig. 4c). Pathway analysis revealed 10 pathways that were significantly associated with the Crohn’s disease phenotype, including the following (Table 1): flagellar assembly, bacterial chemotaxis (cell motility), two-component system (including bacterial chemotaxis), glutathione metabolism, glycine, serine and threonine metabolism, pentose and glucuronate interconversions, glyoxylate and dicarboxylate metabolism, porphyrin and chlorophyll metabolism, synthesis and degradation of ketone bodies, and butanoate metabolism. Butanoate metabolism, flagellar assembly and synthesis and degradation of ketone bodies are consistent with previously observed decreases in short-chain fatty acid metabolism in Crohn’s Disease [21, 22]. Additionally, changes in pathways involved in amino acid and sugar metabolism are consistent with previously observed changes in Crohn’s microbiomes compared to healthy controls [21]. LEfSe was used for a comparison analysis from pathway abundances estimated by HUMAnN. The analysis of the same dataset using HUMAnN and LEfSe predicted 21 differentially abundant pathways, 10 of which overlap with the FMAP analysis or are consistent with previous findings showing changes in amino acid and sugar metabolism (Table 2). On the other hand, ShotgunFunctionalizeR predicted 60 DA pathways, which overlapped well with both HUMAnN and FMAP, and also predicted many pathways in other functional categories, including nucleotide metabolism, translation, and transcription (Additional file 3). These differences can be accounted for because of the differences in the COG database used by ShotgunFunctionalizer versus HUMAnN and FMAP, which relied on KEGG. Using FMAP’s unique operon analysis, by mapping genes to a database of 14,028 known operons [13], our results revealed 16 DA operons involved in flagellar structure and function (Fig. 5).

**Table 1:** FMAP performs pathway analysis of a Crohn’s disease study

Pathway	KO Count	Pathway Coverage	*P*-value
Flagellar assembly	23	0.622	2.977 × 10^−25^
Bacterial chemotaxis	13	0.500	4.331 × 10^−13^
Synthesis and degradation of ketone bodies	2	0.250	2.798 × 10^−2^
Pentose and glucuronate interconversions	8	0.131	9.820 × 10^−4^
Glutathione metabolism	5	0.125	1.077 × 10^−2^
Porphyrin and chlorophyll metabolism	10	0.094	3.110 × 10^−3^
Butanoate metabolism	7	0.085	2.087 × 10^−2^
Glycine, serine and threonine metabolism	7	0.075	3.810 × 10^−2^
Glyoxylate and dicarboxylate metabolism	7	0.074	4.201 × 10^−2^
Two-component system	23	0.054	1.933 × 10^−2^

FMAP can automatically streamline its built-in pathway analysis. Here we show the FMAP pathway analysis results of a Crohn’s disease study (Sequence Read Archive number: SRP002423). KO Count: number of differentially abundant (DA) genes detected by FMAP; Pathway coverage: normalized coverage in each pathway; *P*-value: Fisher’s exact test used to assess the over/under representation of DA genes

**Table 2:** HUMAnN differentially abundant pathways

Pathway	Discriminative Group	log(LDA)	*P*-value
Galactose metabolism	CD	3.238	6.578 × 10^-3^
Streptomycin biosynthesis	CD	3.163	6.578 × 10^-3^
Flagellar assembly	Healthy	2.962	6.578 × 10^-3^
Histidine metabolism	CD	2.581	6.578 × 10^-3^
Flavonoid biosynthesis	Healthy	2.064	6.578 × 10^-3^
Starch and sucrose metabolism	CD	2.945	1.742 × 10^-2^
Tryptophan metabolism	Healthy	2.634	2.460 × 10^-2^
Bacterial chemotaxis	Healthy	2.842	2.725 × 10^-2^
Cell cycle Caulobacter	Healthy	2.830	2.725 × 10^-2^
Nicotinate and nicotinamide metabolism	CD	2.772	2.725 × 10^-2^
Peptidoglycan biosynthesis	CD	2.758	2.725 × 10^-2^
Cyanoamino acid metabolism	Healthy	3.251	3.671 × 10^-2^
Various types of N glycan biosynthesis	Healthy	2.242	3.671 × 10^-2^
Styrene degradation	Healthy	2.017	3.671 × 10^-2^
Malaria	Healthy	2.684	3.722 × 10^-2^
Plant hormone signal transduction	Healthy	2.188	4.118 × 10^-2^
Folate biosynthesis	CD	3.105	4.154 × 10^-2^
Amino sugar and nucleotide sugar metabolism	CD	2.878	4.154 × 10^-2^
Glutathione metabolism	CD	2.623	4.154 × 10^-2^
Aminobenzoate degradation	CD	2.347	4.154 × 10^-2^
Valine leucine and isoleucine degradation	Healthy	2.256	4.154 × 10^-2^

**Fig. 6:**
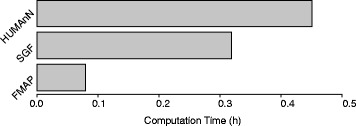
Comparison of computation time in pathway analysis. Barplot of computation time in hours of pathway analysis of HUMAnN, ShotgunFunctionalizeR (SGF), and FMAP. The data of SRP002423 was used to estimate the computation times

Finally, the computation time for metagenomic pathway analysis can be quite burdensome. For example, a dataset of SRP002423 can take up to a half hour to process. Strikingly, FMAP is able to complete a metagenomic pathway analysis five times faster than HUMAnN when analyzing the same data set (Fig. 6). These advantages also hold when compared with the same analysis in ShotgunFunctionalizeR. In all, the computation time for this pathway analysis is quite short for FMAP compared to HUMAnN (Fig. 6).

## Conclusions

Here we introduced FMAP, a straightforward and facile tool for metagenomic/metatranscriptomic functional analysis. FMAP combines read mapping to a reference database, ortholog (KO) quantification and statistical analysis all in one package. The default reference database for FMAP, a functionally annotated filtered UniRef90, is optimized for an increased mapping rate and is updated regularly (server side, every 6 months). Aside from identification of ortholog and pathway analysis, FMAP also has integrated operon analysis. When analyzing identical datasets, FMAP produced results consistent with previously published results and the results of similar software programs, albeit ones that lack FMAP’s mapping or operon analysis features. FMAP results can be used upstream of visualization tools, including the KEGG pathway mapping tool. Finally, FMAP is able to complete these analyses with improved computation time efficiency compared to comparable analysis pipelines. As such, FMAP will have broad appeal and utility for biologists and computational biologists.

## Additional files

Additional file 1: Figure S2. Workflow to create KEGG Filtered UniProt (KFU) Reference Cluster. First, UniProt ID mapping data was downloaded. 80.4 million protein accessions were in the data. To build connections between the UniProt database and KEGG orthology database, we used KEGG LinkDB API (http://www.genome.jp/linkdb/) to select a subset from the UniProt proteins, and to retain only bacteria, archaea or fungi sequences. Next, we built connections between UniProt sequences and UniRef90 sequences via the UniProt ID mapping data, and retain only one-one correspondences. Finally, we obtained 1,995,269 sequences termed as KFU (KEGG filtered UniRef90), and all the sequences had a known relationship between UniRef 90 and KEGG orthology. Solid black lines with arrows indicate data processing steps. Solid black lines with diamond-shaped heads are direct one-to-one relationships. Dashed black lines with diamond-shaped heads are indirect one-to-one relationships. (PDF 33 kb)
Additional file 2: Figure S1. Performance of KO abundance calculation of FMAP and HUMAnN in simulated dataset (454 reads). (A) Heatmap of correlation distances between the true expected, FMAP-predicted and HUMAnN-predicted KO abundances in a simulated dataset (454 reads). (B) Plot of FMAP log2 KO abundance calculated from FMAP compared to log2 KO abundance calculated from HUUMAnN. r is Pearson’s correlation coefficient. (PDF 446 kb)
Additional file 3: Table S1. ShotgunFunctionalizeR predicted 60 differentially abundant pathways. ShotgunFunctionalieR uses quasi-Poisson model to assess each pathway provided in the COG database. Category size, gene family size and adjusted P-value (BH) are provided according to ShotgunFunctionalizeR user’s guide [1]. (DOCX 19 kb)

## Abbreviations

DA: Differentially abundant
FMAP: Functional mapping and analysis pipeline
KEGG: Kyoto encyclopedia of genes and genomes
KO: KEGG ortholog
ODB3: Operon database v3
