# Supplementary figures and images for: FMAP: Functional Mapping and Analysis Pipeline for metagenomics and metatranscriptomics studies
Source: BMC Bioinformatics. 2016 Oct 10;17:420. doi: 10.1186/s12859-016-1278-0 (PMC5057277; doi:10.1186/s12859-016-1278-0)

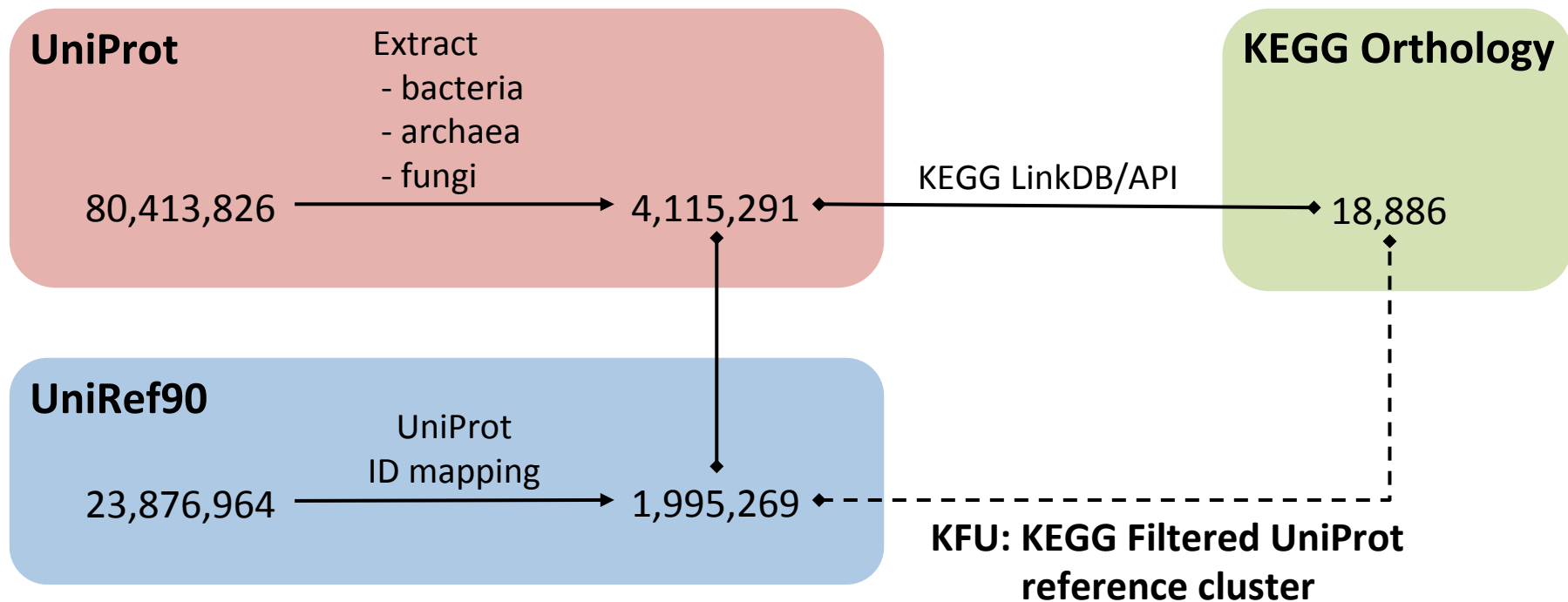

Supplement: Additional file 1: Figure S2. — Workflow to create KEGG Filtered UniProt (KFU) Reference Cluster. First, UniProt ID mapping data was downloaded. 80.4 million protein accessions were in the data. To build connections between the UniProt database and KEGG orthology database, we used KEGG LinkDB API (http://www.genome.jp/linkdb/) to select a subset from the UniProt proteins, and to retain only bacteria, archaea or fungi sequences. Next, we built connections between UniProt sequences and UniRef90 sequences via the UniProt ID mapping data, and retain only one-one correspondences. Finally, we obtained 1,995,269 sequences termed as KFU (KEGG filtered UniRef90), and all the sequences had a known relationship between UniRef 90 and KEGG orthology. Solid black lines with arrows indicate data processing steps. Solid black lines with diamond-shaped heads are direct one-to-one relationships. Dashed black lines with diamond-shaped heads are indirect one-to-one relationships. (PDF 33 kb) [file 12859_2016_1278_MOESM1_ESM.pdf]

A

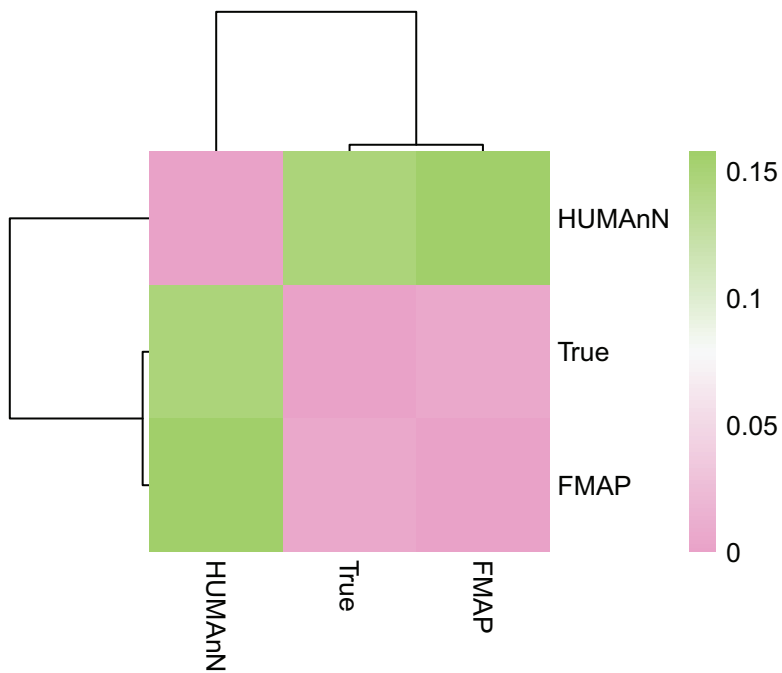

B

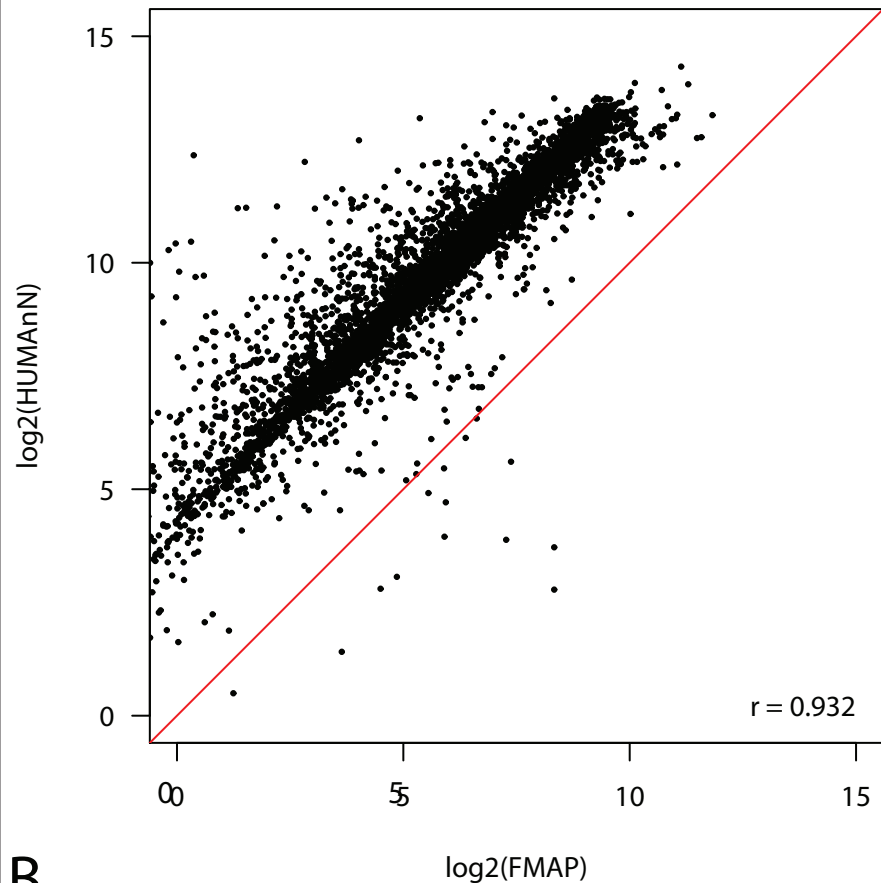

Supplement: Additional file 2: Figure S1. — Performance of KO abundance calculation of FMAP and HUMAnN in simulated dataset (454 reads). (A) Heatmap of correlation distances between the true expected, FMAP-predicted and HUMAnN-predicted KO abundances in a simulated dataset (454 reads). (B) Plot of FMAP log2 KO abundance calculated from FMAP compared to log2 KO abundance calculated from HUUMAnN. r is Pearson’s correlation coefficient. (PDF 446 kb) [file 12859_2016_1278_MOESM2_ESM.pdf]
