# Supplementary material for: FMAP: Functional Mapping and Analysis Pipeline for metagenomics and metatranscriptomics studies
Source: BMC Bioinformatics. 2016 Oct 10;17:420. doi: 10.1186/s12859-016-1278-0 (PMC5057277; doi:10.1186/s12859-016-1278-0)
Supplement: Additional file 3: Table S1. — ShotgunFunctionalizeR predicted 60 differentially abundant pathways. ShotgunFunctionalieR uses quasi-Poisson model to assess each pathway provided in the COG database. Category size, gene family size and adjusted P-value (BH) are provided according to ShotgunFunctionalizeR user’s guide [1]. (DOCX 19 kb) [file 12859_2016_1278_MOESM3_ESM.docx]

Table S1. ShotgunFunctionalizeR predicted 60 differentially abundant pathways. ShotgunFunctionalieR uses quasi-Poisson model to assess each pathway provided in the COG database. Category size, gene family size and adjusted P-value (BH) are provided according to ShotgunFunctionalizeR user’s guide[1].

| Category | Category size | Gene families found | P-value |
| --- | --- | --- | --- |
| Amino acid transport and metabolism | 270 | 270 | 5.18×10^-201^ |
| Transcriptional regulators | 69 | 69 | 2.49×10^-169^ |
| Flagellum structure and biogenesis | 33 | 33 | 2.91×10^-166^ |
| Cell motility | 96 | 96 | 3.24×10^-157^ |
| ×10nergy production and conversion | 258 | 258 | 6.54×10^-132^ |
| Signal transduction mechanisms | 152 | 152 | 1.23×10^-117^ |
| Pyruvate decraboxylation | 7 | 7 | 8.45×10^-113^ |
| Intracellular trafficking, secretion, and | 158 | 158 | 1.89×10^-111^ |
| Defense mechanisms | 46 | 46 | 1.10×10^-98^ |
| Thymidylate biosynthesis | 9 | 9 | 2.40×10^-94^ |
| Cobalamin biosynthesis | 18 | 18 | 2.97×10^-81^ |
| Preprotein translocase subunits | 9 | 9 | 5.29×10^-75^ |
| Secondary metabolites biosynthesis, transport and | 88 | 88 | 7.08×10^-72^ |
| Nucleotide transport and metabolism | 95 | 95 | 1.07×10^-70^ |
| Carbohydrate transport and metabolism | 229 | 229 | 2.01×10^-52^ |
| Biotin biosynthesis | 6 | 6 | 5.07×10^-50^ |
| Lipid A biosynthesis | 9 | 9 | 8.30×10^-48^ |
| Coenzyme transport and metabolism | 179 | 179 | 8.22×10^-44^ |
| Archael/Vacuolar-type H+ ATPase subunits | 9 | 9 | 1.78×10^-43^ |
| Purine biosynthesis | 18 | 18 | 1.18×10^-39^ |
| Transcription | 230 | 230 | 2.05×10^-38^ |
| RNA processing and modification | 25 | 25 | 5.10×10^-33^ |
| Function unknown | 1347 | 1347 | 8.10×10^-33^ |
| Pentose phosphate pathway | 9 | 9 | 2.47×10^-32^ |
| Lipid transport and metabolism | 94 | 94 | 1.46×10^-25^ |
| Methionine biosynthesis | 10 | 10 | 7.54×10^-25^ |
| NA+-transporting NADH:Ubiquinone oxireductase | 7 | 7 | 7.62×10^-25^ |
| Valine biosynthesis | 6 | 6 | 5.94×10^-24^ |
| NADH:Ubiquinone oxidreductase subunits | 15 | 15 | 1.37×10^-22^ |
| Replication, recombination and repair | 238 | 238 | 8.71×10^-22^ |
| TCA cycle | 16 | 16 | 2.46×10^-21^ |
| Histidine biosynthesis | 12 | 12 | 1.33×10^-20^ |
| Cell cycle control, cell division, chromosome | 72 | 72 | 8.61×10^-20^ |
| Riboflavin biosynthesis | 7 | 7 | 7.81×10^-17^ |
| Cytoskeleton | 12 | 12 | 1.07×10^-16^ |
| Trypotophan biosynthesis | 17 | 17 | 4.48×10^-16^ |
| Threonine biosynthesis | 5 | 5 | 7.48×10^-16^ |
| DNA-dependent RNA polymerase subunits | 15 | 15 | 8.94×10^-15^ |
| Gluconeogenesis | 14 | 14 | 1.81×10^-13^ |
| Phenylalanine/tyrosine biosynthesis | 14 | 14 | 4.25×10^-13^ |
| Basal transription factors | 11 | 11 | 8.92×10^-13^ |
| FAD biosynthesis | 9 | 9 | 4.33×10^-12^ |
| Fatty acid biosyntheis | 13 | 13 | 1.47×10^-11^ |
| Glycolysis | 14 | 14 | 2.06×10^-9^ |
| Inorganic ion transport and metabolism | 212 | 212 | 7.02×10^-9^ |
| Ribosomal proteins - large subunit | 51 | 51 | 3.98×10^-8^ |
| Menaquinone biosynthesis | 16 | 16 | 8.77×10^-8^ |
| Proline biosynthesis | 5 | 5 | 2.74×10^-7^ |
| Heme biosynthesis | 14 | 14 | 1.69×10^-5^ |
| NAD biosynthesis | 7 | 7 | 3.99×10^-5^ |
| Isoleucine biosynthesis | 6 | 6 | 6.70×10^-5^ |
| Pyridoxal phosphate biosynthesis | 8 | 8 | 8.17×10^-5^ |
| Arginine biosynthesis | 11 | 11 | 1.48×10^-4^ |
| Pyrimidine biosynthesis | 14 | 14 | 1.94×10^-4^ |
| Translation, ribosomal structure and biogenesis | 245 | 245 | 4.57×10^-4^ |
| DNA polymerase III subunits | 8 | 8 | 1.28×10^-3^ |
| Leucine biosyntehsis | 10 | 10 | 5.63×10^-3^ |
| Ubiquinone biosynthesis | 15 | 15 | 9.24×10^-3^ |
| F0F1-type ATP synthase subunits | 9 | 9 | 1.28×10^-2^ |
| Thiamine biosynthesis | 10 | 10 | 2.07×10^-2^ |

1. Kristiansson E, Hugenholtz P, Dalevi D: **ShotgunFunctionalizeR: an R-package for functional comparison of metagenomes**. *Bioinformatics* 2009, **25**(20):2737-2738.
